# PTH stimulation of *Rankl* transcription is regulated by SIK2 and 3 and mediated by CRTC2 and 3 through action of protein phosphatases 1, 2, 4, and 5

**DOI:** 10.1016/j.jbc.2025.110434

**Published:** 2025-07-01

**Authors:** Michael J. Mosca, Zhiming He, Nagarajan Selvamurugan, Jobin Joseph, Whitney Petrosky, Carole Le Henaff, Nicola C. Partridge

**Affiliations:** 1 Department of Molecular Pathobiology, New York University College of Dentistry, New York, New York, USA; 2 Vilcek Institute of Graduate Biomedical Sciences, New York University School of Medicine, New York, New York, USA; 3 Department of Biotechnology, School of Bioengineering, SRM Institute of Science and Technology, Kattankulathur, Tamil Nadu, India; 4 Center for Advanced Biotechnology and Medicine, Rutgers University, Piscataway, New Jersey, USA

**Keywords:** osteoblast, parathyroid hormone, osteoporosis, CREB-regulated transcriptional coactivators, phosphoprotein phosphatases, salt-inducible kinases, receptor activator of nuclear factor kappa-β ligand, nuclear translocation

## Abstract

Osteoporosis is characterized by a decrease in the density and quality of bone tissue and is associated with substantial morbidity/mortality. Homeostatic processes that form new and remove old/damaged bone are dysregulated, with resultant net bone resorption. Parathyroid hormone (PTH) is a key regulator of this homeostasis and along with its analogs has been used to treat osteoporosis, however its use is limited to an “anabolic window”. PTH stimulates both formation and resorption, the latter largely due to increased receptor activator of nuclear factor kappa-β ligand (RANKL). Our laboratory has found a cascade of messengers, Salt-inducible kinases (SIKs) and protein phosphatases (PPs), regulate nuclear translocation of CREB-regulated transcriptional coactivators (CRTCs), but the individual and/or combined contributions of these factors has not yet been established in osteoblasts. In this study, we reveal precise mechanisms involved in CRTC1/2/3 nuclear translocation and delineate their roles as co-activators of *Tnfsf11* (RANKL gene name) transcription throughout osteoblast differentiation using a primary mouse calvarial osteoblast model. By performing a series of siRNA knockdowns of CRTC1/2/3, SIK1/2/3, and PP1/2/3/4/5/6/7, we determined the regulation of CRTCs upon PTH-stimulation *via* qPCR, quantitative immunofluorescence, Western blotting, and co-immunoprecipitation. CRTC2 is determined to be the primary co-activator of *Tnfsf11* transcription with SIK2/3 inhibition upon PTH-stimulation making CRTC2 available for nuclear translocation by PP1/2/4/5 action. Understanding the mechanisms involved in this cascade may reveal novel targets in the treatment of osteoporosis and allow researchers a new line of approach for drug design that could overcome the “anabolic window” limiting current PTH-derived treatments.

Osteoporosis is a prevalent disease of lack of sex steroids and aging characterized by a decrease in the density and quality of bone tissue and is associated with substantial morbidity/mortality (1). In osteoporosis, the homeostatic processes that form new and remove old/damaged bone are dysregulated, resulting in excessive resorption (2). Parathyroid hormone (PTH) is a key regulator of bone homeostasis and along with its analogs has been used to treat osteoporosis (3). Although PTH has positive anabolic effects on bone it can also stimulate catabolism, the latter largely through activity of receptor activator of nuclear factor kappa-β ligand (RANKL) (4). Treatment of osteoporosis with PTH (1–34) is limited by an “anabolic window,” after which biochemical markers of resorption increase, lessening the anabolic effect (5, 6). Theoretically, it may be possible to retain just the positive effects of PTH-derived treatments if transcription of *TNFSF11* (RANKL gene name) can be inhibited.

RANKL is a pro-osteoclastogenic cytokine with roles in crosstalk between osteoblasts/osteoclasts and is the primary source of PTH-induced bone resorption through differentiation and stimulation of osteoclasts (7). RANKL, produced by osteoblasts, osteocytes, and bone marrow stromal cells, binds to its receptor, RANK, which is present on the surface of osteoclast precursors. This, together with macrophage-colony stimulating factor (M-CSF), induces the differentiation of monocyte-lineage cells into osteoclasts (8). Osteoprotegerin (OPG) functions by binding RANKL, preventing RANKL from interacting with RANK, thereby inhibiting the formation, function, and survival of osteoclasts (9). This process helps to maintain a balance between the activity of osteoclasts and the activity of osteoblasts.

PTH and its analogs bind the same receptor on osteoblasts, PTHR1, activating a signaling cascade leading to *Tnfsf11* transcription (10, 11). Recent work has implicated a cascade of messengers, enzymes, kinases, and phosphatases in regulating potential coactivators of *Tnfsf11* transcription, known as CREB-regulated transcriptional coactivators (CRTCs) (10). The CRTC family is a group of transcriptional coactivators that modulate the activity of various transcription factors in response to cellular signaling events, particularly the cAMP signaling pathway (12, 13). There are three isoforms: CRTC1, CRTC2, and CRTC3. Under basal conditions, CRTCs are highly phosphorylated and maintained in the cytoplasm *via* interactions with 14-3-3 proteins (14). Increases in cAMP and calcium trigger CRTC dephosphorylation and release from 14-3-3 complexes. CRTCs then translocate into the nucleus, where they bind unknown bZIP transcription factor(s), which activate *Tnfsf11* transcription (10). Our laboratory’s current model implicates several salt-inducible kinases (SIKs) and protein phosphatases (PPs) with CRTC regulation; however, their individual contributions/interactions need to be elucidated.

The goal of this study was to determine the roles of each CRTC throughout osteoblast differentiation with respect to *Tnfsf11* transcription and to elucidate the specific mechanisms that lead to regulation of the key CRTCs upon PTH (1–34) treatment using primary calvarial osteoblasts. Factors that were investigated included CRTC1, CRTC2, CRTC3, SIK1, SIK2, SIK3, PP1, PP2 (aka PP2A), PP3 (aka PP2B/Calcineurin), PP4, PP5, PP6, and PP7. We determined the individual/combined roles of these factors on CRTC nuclear translocation and *Tnfsf11* expression upon PTH-treatment. These data reveal novel targets that could theoretically ameliorate dysregulation in diseased tissue with excessive *Tnfsf11* expression/activity.

## Results

### The primary co-activator in osteoblastic cells for Tnfsf11 (Rankl) transcription is CRTC2 with CRTC3 being compensatory

Current models suggest that PTH-induced stimulation of PTHR1 leads to CRTC dephosphorylation, subsequent nuclear translocation, and activation of *Tnfsf11* transcription (10). Studies from an osteocytic cell line have suggested CRTC2 is the primary co-activator for PTH-induced *Tnfsf11* transcription (15, 16) and initial data from our laboratory suggested CRTC3 was the primary co-activator of *Tnfsf11* transcription (10) but an examination of all three CRTCs had not been fully conducted in primary osteoblast cells. These data suggested there might be a switch in CRTC involvement for transcriptional initiation from progenitors → mature → mineralized osteoblasts. We hypothesized that CRTC3 would be the primary co-activator in undifferentiated and mature osteoblasts, switching to CRTC2 in mineralized osteoblasts.

**Figure 1:**
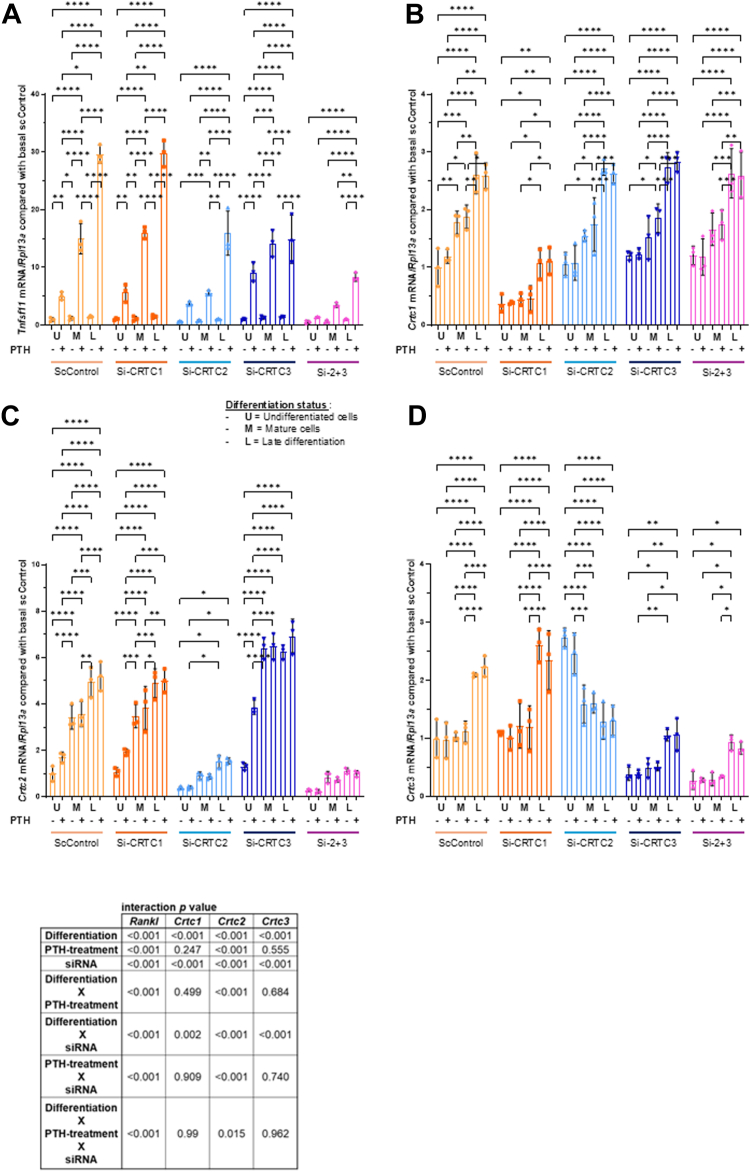
**CRTC1, CRTC2, and CRTC3 as *Tnfsf11* transcriptional co-activators in mouse calvarial osteoblasts throughout differentiation.** Primary calvarial osteoblasts were plated according to three differentiation protocols: (1) undifferentiated, U, (5 days to reach confluence in αMEM media), (2) mature, M, (5 days to reach confluence + 2 days in osteogenic differentiation media), and (3) late stage mineralized, L, (5 days to reach confluence + 7 days in osteogenic differentiation media). For all time points siRNA transfection (5 μl of lipofectamine + 30 pmol of siRNA in 1 ml of medium) occurred 48 h prior to treatment with 10 nM of PTH (1–34) for 4 h. Efficacy of knockdowns are shown in the Western blots in Figure 2*B*. Cells were then harvested for RNA and cDNA synthesized for qRT-PCR analyses of *Tnfsf11*, *Crtc1*, *Crtc2*, and *Crtc3* mRNAs at each differentiation stage. Each individual data point represents a separate experiment in which the value was averaged from technical triplicates of three wells. Standard deviation is calculated using the averaged value over three experiments. Therefore, results are shown as mean ± SD where n = 3. Data represent mRNA normalized to the housekeeping gene (*Rpl13a*) compared with the same amount of lipofectamine and concentration of scrambled siRNA for the undifferentiated basal samples, ∗*p* < 0.05, ∗∗*p* < 0.01, ∗∗∗*p* < 0.001. Three-way ANOVA was used to obtain the interaction *p* values followed by 2-way ANOVA with *post hoc* Tukey tests for each differentiation status.

To determine the roles of each CRTC throughout osteoblast differentiation, we evaluated *Crtc1*, *Crtc2*, *Crtc3*, and *Tnfsf11* mRNAs using the protocols for undifferentiated, mature, and late-stage mineralized osteoblasts (Fig. 1). Data show a significant increase in PTH-stimulated *Tnfsf11* mRNA abundance as cells mature. Additionally, siRNA knockdowns did not affect basal *Tnfsf11* levels (detailed explanation can be found in, Figs. S1–S3).

The knockdown of CRTC1 did not lead to any significant changes in *Tnfsf11* mRNA at any stage of differentiation when compared to si-scrambled controls (Fig. 1*A*). Knockdown of CRTC2 reduced PTH-stimulated *Tnfsf11* mRNA in undifferentiated osteoblasts but was not significant; CRTC2 knockdown significantly reduced PTH-stimulated *Tnfsf11* mRNA in both mature and mineralized osteoblasts compared to controls. CRTC3 knockdown significantly increased PTH-stimulated *Tnfsf11* mRNA in undifferentiated osteoblasts, had no effect in mature osteoblasts, and significantly decreased PTH-stimulated *Tnfsf11* mRNA in mineralized osteoblasts. Combined knockdown of CRTC2+CRTC3 significantly reduced PTH-stimulated *Tnfsf11* mRNA at all three time points compared to controls and to individual CRTC2/3 knockdowns.

Knockdowns of CRTC2, CRTC3, or CRTC2+3 did not result in changes in CRTC1; CRTC1 knockdown caused up to ∼60% reduction in *Crtc1* mRNA at all stages (Fig. 1*B*). Knockdown of CRTC3 significantly increased *Crtc2* mRNA at all three stages compared with controls (Fig. 1*C*), and knockdown of CRTC2 significantly increased *Crtc3* mRNA in undifferentiated cells by ∼150% but decreased it by ∼45% in mature osteoblasts. Combined knockdown of CRTC2+3 led to further reduction in both *Crtc2* and *Crtc3* mRNAs at all stages of differentiation compared to individual CRTC2/3 knockdowns (Fig. 1, *C* and *D*). All three *Crtcs* demonstrated an increase in basal expression with differentiation.

**Figure 2:**
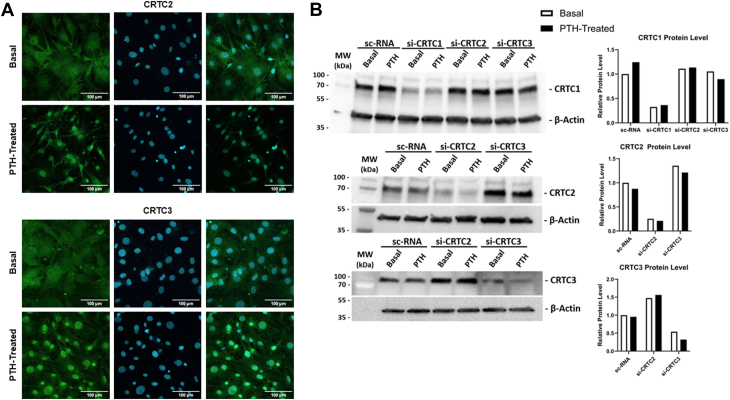
**Immunofluorescent staining and protein compensation of CRTC isoforms in mouse calvarial osteoblasts.***A*, primary calvarial osteoblasts were plated and differentiated according to the mature protocol (5 days to reach confluence + 2 days in osteogenic differentiation media) without the addition of siRNAs. They were then treated with 10 nM of PTH for 45 min and subsequently fixed/stained with CRTC2 and CRTC3 antibodies for immunofluorescent imaging or DAPI for nuclei. Images were obtained by confocal microscopy as described in the Methods. *Left panels*, CRTC2 or 3, *middle panels* DAPI, *right panels*, overlay. *B*, primary osteoblasts were plated according to the mature protocol in 100 mm dishes with siRNA transfection (30 μl of lipofectamine + 40 nM of siRNA in 6 ml of medium) 48 h prior to treatment with 10 nM of PTH (1–34) for 4 h. Western blots of CRTC siRNA knockdowns were conducted with CRTC1, CRTC2, CRTC3, and β-actin antibodies to confirm siRNA knockdowns decreased protein levels and to determine if any compensation occurred among the isoforms. Quantification of the Western blots is shown on the *right panels*; statistics cannot be provided for these data.

We then examined if CRTC translocation could be visualized/quantified *via* immunofluorescent staining (Fig. 2*A*). We observed that both CRTC2 and CRTC3 clearly translocated from the cytoplasm to the nucleus upon PTH treatment, with ∼40% of nuclei staining for CRTC2 in the basal state and ∼90% of nuclei in PTH-treated samples. Very little nuclear staining was observed for CRTC3 in the basal state, but it was present in ∼90% of nuclei in PTH-treated samples.

Examining these data suggests there are changes with respect to the roles of each CRTC from osteogenic progenitors to mineralized late-stage osteoblasts. CRTC1 plays no role in *Tnfsf11* transcription. CRTC2 appears to be the most consistent co-activator of *Tnfsf11* transcription throughout all stages of differentiation, with CRTC3 being redundant, switching to equal involvement in mineralized osteoblasts. There also appears to be cross-regulation occurring between the factors since knocking down one causes an increase in the other (Fig. 1, *C* and *D*). Examination of CRTC2 and CRTC3 proteins from additional siRNA knockdowns shows that the mRNA cross-regulation we observed also results in changes in protein: knockdown of CRTC2 increased CRTC3 protein levels, and knockdown of CRTC3 increased CRTC2 protein levels (Fig. 2*B*).

Our time course data provided valuable insight into how we would move forward with our experiments. Both the undifferentiated and mineralized time points came with difficulty in transfection: undifferentiated osteoblasts would easily die upon transfection, and mineralized osteoblasts required higher doses of lipofectamine + siRNA and would not always produce effective knockdowns due to the post-mitotic state of the cells and their mineralized nodules. Due to these findings, having no issues with the 5 +2 days protocol in terms of cell viability, transfection, and this differentiation stage most accurately representing the expression profile of the cells we were interested in, we decided to utilize the mature osteoblast differentiation protocol for the rest of the experiments. For additional information on how we determined protocols, see (Figs. S1–S3).

### SIK2 and 3 knockdowns mimic PTH-treatment; SIK1 appears not to be involved; inhibition mimics PTH action

SIKs are a sub-family of serine/threonine protein kinases that belong to the AMP-activated protein kinase (AMPK) family that consists of three kinases (SIK1, SIK2, and SIK3) (17). SIK cellular activity is tonically ‘on’ due to constitutive LKB1-mediated phosphorylation, and a key role is to regulate the changes in phosphorylation status of class IIa HDACs and CRTCs (18, 19, 20). SIK-mediated phosphorylation of class IIa HDAC and CRTC proteins leads to their cytoplasmic retention and latent inactivation (21, 22, 23). Upon inhibition by PKA activity, SIKs dissociate from their substrates, allowing the substrates to be dephosphorylated and to translocate into the nucleus to regulate gene expression. Increased cAMP levels due to PTH-treatment lead to PKA-mediated SIK inhibition, reducing SIK cellular activity and therefore their role in the sequestration of their targets.

**Figure 3:**
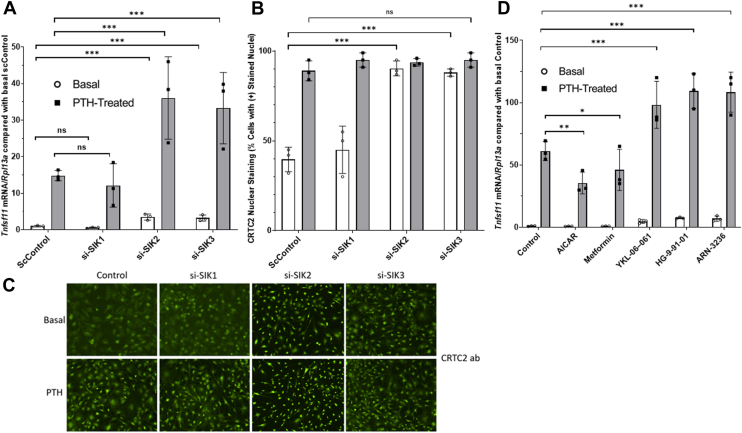
**Effects of SIK siRNA knockdown, inhibition, and activation on *Tnfsf11* expression and CRTC2 nuclear translocation in mouse calvarial osteoblasts.** Primary calvarial osteoblasts were plated for 5 days to reach confluence + 2 additional days in osteogenic differentiation media. *A–C*, cells were treated with siRNAs (40 nM) for SIKs 48 h prior to treatment with 10 nM PTH (1–34) (4 h for mRNA, 45 min for immunofluorescence). Efficacy of knockdowns is shown in (Figs. S5 and S6). *A*, *Tnfsf11* mRNA after SIK1/2/3 knockdowns. *B*, CRTC2 nuclear staining after SIK1/2/3 knockdowns. *C*, Representative CRTC2 immunofluorescent images after siRNA knockdowns of SIK1/2/3. *D*, primary calvarial osteoblasts were plated and allowed to differentiate according to the mature protocol but no transfection was performed. After differentiating, the cells were treated with 10 nM PTH (1–34) for 4 h. At the same time these cells were additionally supplemented with either 1 mM AICAR, 10 mM metformin, 50 nM YKL-06-061, 50 nM HG-9-91-01, or 50 nM ARN-3236 for 4 h prior to harvest and analysis of *Tnfsf11* mRNA *via* qRT-PCR. Each individual data point represents a separate experiment in which the value was averaged from technical triplicates of three wells. Standard deviation is calculated using the averaged value over three experiments. Therefore, results are shown as mean ± SD where n = 3. Data represent *Tnfsf11* normalized to *Rpl13a* and compared with basal control samples. ∗*p* < 0.05, ∗∗*p* < 0.01, ∗∗∗*p* < 0.001 Two-way ANOVA with *post hoc* Tukey tests used.

**Figure 4:**
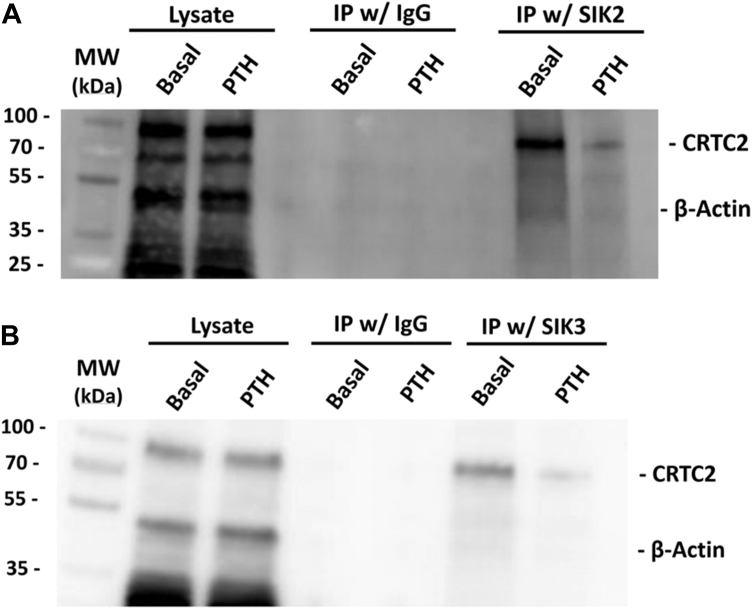
**SIKs and CRTC2 co-immunoprecipitate in mouse calvarial osteoblasts.** Osteoblasts were plated using the mature differentiation protocol and subsequently treated with or without 10 nM PTH (1–34) for 15 min. Immunoprecipitations were conducted using SIK2 (*A*) and SIK3 (*B*) antibodies, membranes were subsequently immunoblotted for CRTC2 and β-actin.

To determine the roles of each SIK on CRTC2 regulation and *Tnfsf11* transcription, we evaluated *Tnfsf11* mRNA, CRTC2 nuclear staining (Fig. 3, *A*–*C*), and utilized co-immunoprecipitation to confirm potential interactions between SIKs and CRTCs after siRNA knockdowns of SIK1, SIK2, and SIK3 in basal and PTH-treated samples (Fig. 4). We hypothesized that siRNA knockdowns of the SIKs would reduce CRTC2 cytoplasmic sequestration, allowing CRTC2 to translocate into the nucleus and activate *Tnfsf11* transcription.

There were no significant differences between si-scrambled controls and SIK1 knockdown in *Tnfsf11* mRNA expression or CRTC2 nuclear translocation under either basal or PTH-treated states (Fig. 3, *A* and *B*). There is a significant increase in *Tnfsf11* mRNA and CRTC2 translocation between the basal states of SIK2/3 siRNA knockdowns and controls. Similarly, PTH-treated cells with siRNA knockdowns of SIK2/3 show a significant increase in *Tnfsf11* mRNA compared with PTH-treated controls, suggesting SIK2 and SIK3 knockdown not only mimic PTH-treatment but also exacerbate it. This further increase in *Tnfsf11* expression does not appear to be a 1:1 result of an increase in CRTC2 translocation as there are no significant differences between the positively stained nuclei in PTH-treated samples of si-controls and untreated siRNA knockdowns of SIK2/3 (Fig. 3, *B* and *C*). In other words, knockdown of SIKs alone causes maximal CRTC2 translocation to the nucleus but other events must increase *Tnfsf11* transcription.

Additional wild-type samples were harvested in basal and PTH-treated conditions to assess physical interactions *via* co-immunoprecipitation (Fig. 4). Samples were immunoprecipitated with an antibody to CRTC2, and immunoblotted for SIK2/3, and additional samples were immunoprecipitated with an antibody to SIK2/3 and immunoblotted for CRTC2. Both show substantial interaction among SIKs with CRTC2 in the basal state, which is mostly lost upon treatment with PTH (1–34).

The data above show that basal SIK2/3 activity causes CRTC2 cytoplasmic sequestering, which is lost upon PTH-treatment or by siRNA knockdown of SIK2/3. These data led us to hypothesize that small-molecule activators of SIKs would reverse this unwanted signaling in osteoblasts and lead to reduced PTH-stimulated *Tnfsf11* mRNA. We tested our PTH-treatment model in wild-type calvarial osteoblasts with supplementation with metformin (shown previously to have effects on bone *in vivo* (24) and to decrease *Tnfsf11 in vitro* (25) and is thought to be an AMPK activator), AICAR (5-aminoimidazole-4-carboxamide ribonucleotide, an AMP analog and AMPK activator), and SIK inhibitors (17) (YKL-06–061, HG-9-91–01, and ARN-3236).

*Tnfsf11* mRNA was determined in basal and PTH-treated states (Fig. 3*D*). Data show a modest reduction in *Tnfsf11* mRNA in the PTH-treated state with AICAR and metformin. All three SIK inhibitors caused a significant increase in *Tnfsf11* in the PTH-treated state compared to controls, enhancing the PTH effect and a tendency to an increase in the basal state, but this did not reach significance.

### Individual knockdowns of catalytic subunits for serine-threonine protein phosphatases show that Tnfsf11 mRNA abundance and CRTC2 nuclear translocation are linked to their activity

Data so far show SIK2 and SIK3 regulate the abundance of CRTC2 in the cytoplasm, but additional interaction is required to remove phosphate group(s) on CRTC2 to allow it to translocate into the nucleus. The phosphoprotein phosphatase family of protein serine/threonine phosphatases (26) is a multimeric protein complex consisting of a catalytic subunit and one or two scaffold/regulatory subunits and has been implicated in the regulation of CRTCs through dephosphorylation (20, 27). Representative members of the PP family include PP1, PP2A, PP2B/PP3 (commonly known as calcineurin), PP4, PP5, PP6, and PP7. Several PPPs have been confirmed to aid in dephosphorylating CRTCs (28, 29), allowing them to translocate into the nucleus and bind a bZIP transcription factor and activate transcription of target genes. A recent study from our laboratory showed that okadaic acid (OA), a potent inhibitor of both PP1 and PP2A, significantly reduced CRTC translocation upon PTH-treatment compared to controls, suggesting PP1/2 and/or other PPs from this family may dephosphorylate CRTC2 once disassociated from 14-3-3 in the cytoplasm (10).

**Figure 5:**
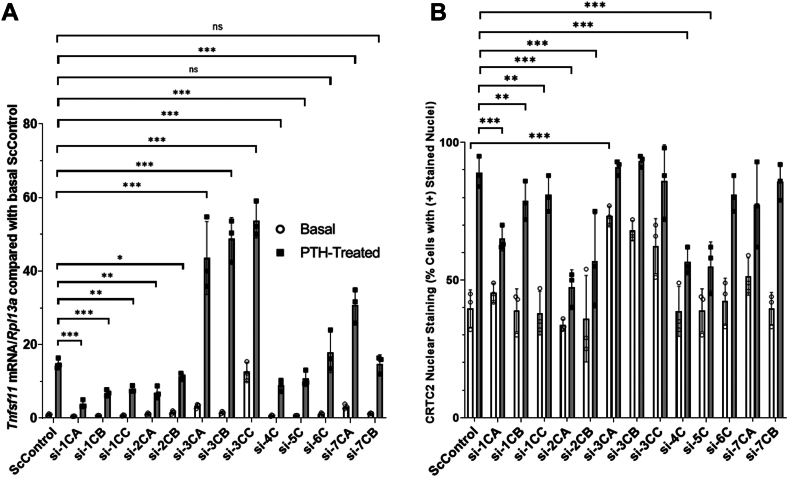
***Tnfsf11* expression and CRTC2 nuclear staining upon siRNA knockdown of individual catalytic subunits for each Serine/Threonine Protein Phosphatase.** Primary calvarial osteoblasts were plated for 5 days to reach confluence + 2 additional days in osteogenic differentiation medium. siRNAs of each individual catalytic subunit of PPs1-7 (40 nM) were added 48 h prior to treatment with 10 nM PTH (1–34) for 4 h for mRNA or 45 min for immunofluorescence. Efficacy of knockdowns is shown in (Figs. S5 and S6). Cells were subsequently harvested to analyze *Tnfsf11* mRNA *via* qRT-PCR or fixed to assess levels of CRTC2 staining in the cytoplasm and nucleus of basal and PTH-treated samples. *A*, data represent *Tnfsf11* normalized to *Rpl13a* and compared with basal ScControl samples. *B*, CRTC2 nuclear staining after individual catalytic unit knockdowns. Each individual data point represents a separate experiment in which the value was averaged from technical triplicates of three wells. Standard deviation is calculated using the averaged value over three experiments. Therefore, results are shown as mean ± SD where n = 3. ∗*p* < 0.05, ∗∗*p* < 0.01, ∗∗∗*p* < 0.001. Two-way ANOVA with *post hoc* Tukey tests used.

To parse out the role each PP may have in the regulation of CRTC2 nuclear translocation and *Tnfsf11* expression, we first performed siRNA knockdowns of each catalytic subunit(s) for all serine/threonine PPs and determined resultant *Tnfsf11* mRNA and CRTC2 translocation by qRT-PCR and quantitative immunofluorescence (Fig. 5). If a subunit is responsible for CRTC2 regulation upon SIK inhibition, we expect to see reduced *Tnfsf11* mRNA and CRTC2 in the nucleus compared to controls. We hypothesized that knockdowns of PP1 and/or PP2 would prevent CRTC2 dephosphorylation, subsequent translocation, and therefore less *Tnfsf11* transcripts due to previous data suggesting their direct involvement. We also hypothesized that knockdowns of PP3/4/5/6/7 may yield comparable results if additionally involved.

Knockdown of all catalytic subunits of PP1, PP2, PP4, and PP5 resulted in significant decreases in PTH-treated *Tnfsf11* mRNA compared to controls (Fig. 5*A*). Knockdown of calcineurin (PP3) and PP7CA significantly increased both basal and PTH-treated *Tnfsf11* compared to controls; knockdown of PP6C and PP7CB did not lead to changes in either basal or PTH-treated states. CRTC2 nuclear translocation shows analogous data: PP1, 2, 4, and 5 show involvement in reducing the effectiveness of PTH-treatment (Fig. 5*B*).

### Combination knockdowns show PPs are integral to PTH-induced Tnfsf11 transcription

To create the most effective reduction in PTH-treatment’s effectiveness on *Tnfsf11* transcription, we incorporated combined knockdown of each PP and then a cocktail including all PPs that reduce *Tnfsf11* mRNA and CRTC2 translocation (si-PP1CA+1CB+1CC together for total knockdown of PP1, si-PP2CA + PP2CB for a total PP2 knockdown, followed by complete PP1+PP2+PP4+PP5 knockdown).

**Figure 6:**
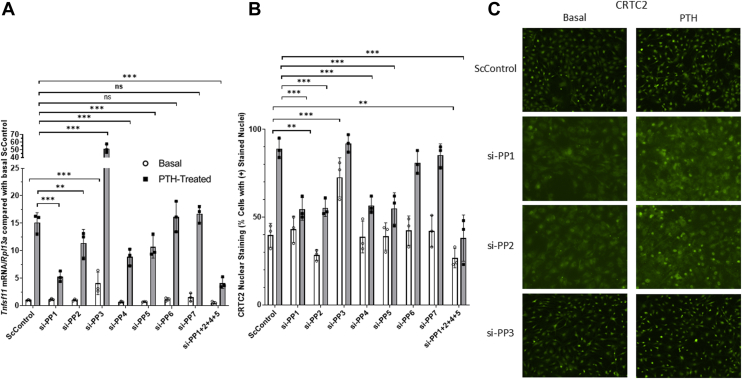
**Role of Serine/Threonine Protein Phosphatases 1 to 7 on PTH-induced *Tnfsf11* transcription in mouse calvarial osteoblasts.** Primary calvarial osteoblasts were plated for 5 days to reach confluence + 2 additional days in osteogenic differentiation medium. siRNAs (40 nM for each catalytic subunit, up to 280 nM for si-PP1+2+4+5, no significant difference was present between 40 nM and 280 nM si-scrambled controls, see Fig. S2 for additional explanation) were added 48 h prior to treatment with 10 nM PTH (1–34) (4 h for mRNA and 45 min for immunofluorescence). *A*, *Tnfsf11* mRNA after PP1-7 knockdowns and a combined cocktail knockdown for PP1/2/4/5. *B*, CRTC2 nuclear staining after PP1-7 knockdowns and a combined cocktail for PP1/2/4/5. Efficacy of knockdowns is shown in (Figs. S5 and S6). Each individual data point represents a separate experiment in which the value was averaged from technical triplicates of three wells. Standard deviation is calculated using the averaged value over three experiments. Therefore, results are shown as mean ± SD where n = 3. Data represent *Tnfsf11* normalized to *Rpl13a* and compared with basal ScControl samples, ∗*p* < 0.05, ∗∗*p* < 0.01, ∗∗∗*p* < 0.001. Two-way ANOVA with *post hoc* Tukey tests used. *C*, representative CRTC2 immunofluorescent images for siRNA knockdowns of PP1/2/3.

Full knockdown of PP1 catalytic subunits significantly reduced PTH-treated *Tnfsf11* mRNA and CRTC2 translocation compared to individual catalytic subunits alone (Fig. 6, *A* and *B*). Similarly, total knockdown of PP3 shows enhanced amplification of basal and PTH-treated *Tnfsf11* mRNA; however, quantitative immunofluorescence data is comparable to individual knockdowns due to percentages of basal and PTH-treated CRTC2 in the nucleus already reaching >90% (Fig. 6*C*). A combined knockdown of PP1+PP2+PP4+PP5 catalytic subunits reduced PTH-stimulated *Tnfsf11* mRNA and CRTC2 translocation significantly more than any individual prior knockdown. A summary of the effects of SIK and PP knockdown on *Tnfsf11* mRNA and CRTC2 nuclear staining can be found in Table 1.

**Table 1:** Summarized results of SIK and PP knockdowns

Knockdown	*Tnfsf11* mRNA	CRTC2 nuclear staining
SIK 1	No change	No change
SIK 2	↑Basal ↑PTH-treated	↑Basal
SIK 3	↑Basal ↑PTH-treated	↑Basal
PP1	↓PTH-treated	↓PTH-treated
PP2	↓PTH-treated	↓Basal ↓PTH-treated
PP3	↑Basal ↑PTH-treated	↑Basal
PP4	↓PTH-treated	↓PTH-treated
PP5	↓PTH-treated	↓PTH-treated
PP6	No change	No change
PP7	No change	No change
PP1+2+4+5	↓↓Basal ↓↓PTH-treated	↓↓Basal ↓↓PTH-treated

**Figure 7:**
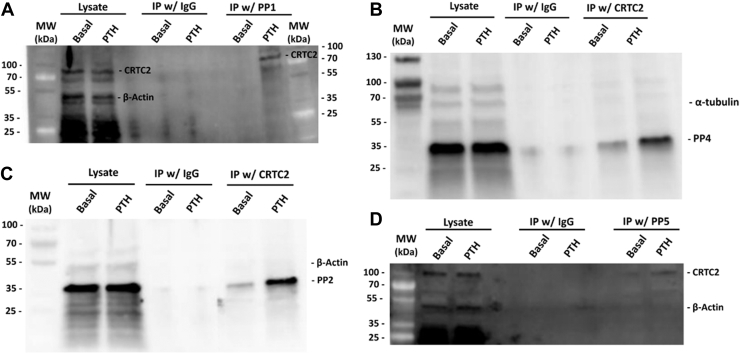
**PP1/2/4/5 and CRTC2 co-immunoprecipitate in mouse calvarial osteoblasts.** Osteoblasts were plated in 100 mm dishes and allowed to differentiate using the established protocol. Cells were treated with 10 nM PTH (1–34) for 15 min prior to harvest. Immunoprecipitations were conducted using CRTC2 and immunoblotted for PP1/2/4/5 or IP with PP1/2/4/5 and immunoblotted for CRTC2.

Additional cell samples were harvested under basal and PTH-treated conditions to assess physical interactions by co-immunoprecipitation (Fig. 7). Samples were immunoprecipitated with CRTC2 antibody and immunoblotted for PPs (Fig. 7, *B* and *C*) or immunoprecipitated with PP antibodies and immunoblotted for CRTC2 (Fig. 7, *A* and *D*). There are lower interactions among PPs and CRTC2 in the basal state, which is increased upon treatment with PTH (1–34). These interactions may therefore facilitate dephosphorylation and subsequent translocation of CRTC2 into the nucleus upon cAMP signaling after PTH-treatment.

## Discussion

**Figure 8:**
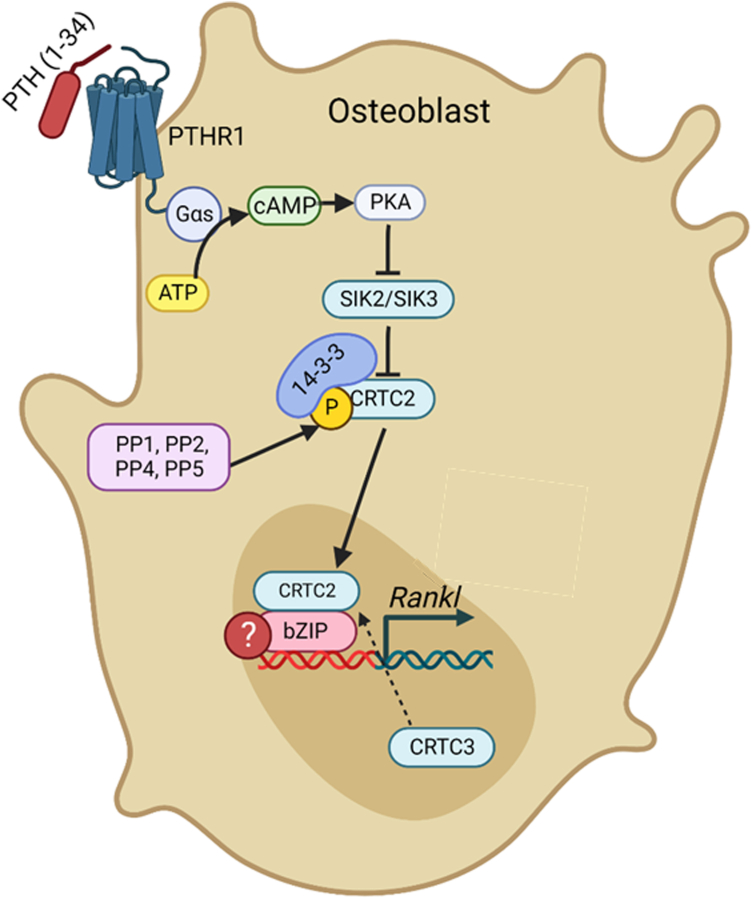
**Model of PTH-induced *Tnfsf11* transcription in primary mouse osteoblasts.** Data from this paper delineated more precise roles of SIKs and PPs in this mechanism. (1) SIK1, PP6, and PP7 are not involved, (2) SIK2/3 cause CRTC2 to be sequestered in the cytoplasm in the basal state, which is lost upon PTH-treatment, (3) PP1, 2, 4, and 5 appear to act to remove phosphates from CRTC2 allowing it to translocate into the nucleus.

The data presented in this study delineated more intricate signaling of PTH-induced *Tnfsf11* transcription in osteoblasts and led to the model shown in Figure 8. Prior to the research conducted here we knew PTH binds PTHR1 in osteoblasts, activating a signaling cascade leading to *Tnfsf11* transcription through a cascade of messengers, enzymes, kinases, and phosphatases to regulate CRTC translocation into the nucleus to activate *Tnfsf11* transcription, but we did not know the detailed mechanism in the osteoblast (10).

Data presented demonstrate the pivotal roles of CRTC2 and CRTC3 in *Tnfsf11* transcription in osteoblasts after PTH-treatment; CRTC1 does not appear to be involved. Notably, our findings contradicted our initial hypothesis that CRTC3 would be the primary co-activator, with a switch to CRTC2 in mineralized osteoblasts. In the undifferentiated state, it initially appears that individual knockdown of CRTCs does not affect PTH-stimulated *Tnfsf11* transcription in osteoblasts, since the reduction is non-significant for CRTC2 knockdown and CRTC3 knockdown causes a significant increase in PTH-stimulated *Tnfsf11* mRNA. Further examination shows the compensation among the CRTCs is most prevalent at this stage of differentiation and that CRTC2 is the primary co-activator. *Crtc2* mRNA is upregulated ∼2-fold upon CRTC3 knockdown and knockdown of CRTC2+3 decreases *Tnfsf11* by ∼80% despite neither CRTC2/3 individually leading to reduced *Tnfsf11*. A similar but not the same pattern occurs in mature osteoblasts. The primary difference is that CRTC3 knockdown does not affect *Tnfsf11*, while CRTC2 knockdown does cause significant reduction. In mineralized osteoblasts CRTC2 and CRTC3 appear to have equal roles in co-activation of PTH-stimulated *Tnfsf11* transcription both individually and an increased reduction when both are knocked down.

The difference between the roles of CRTCs may also be attributed to small differences in their primary sequences, leading to distinct interactions (or lack thereof) with 14-3-3 proteins and differing reactions to external stimuli (30). Recent work has uncovered a location within the primary sequence of the CRTC isoforms where there are three serine-rich areas responsible for interacting with transcription factors like CREB as well as regulatory regions that control their subcellular localization and activity (30). These regions also facilitate interactions with 14-3-3 and uncovered that one of the regions utilizes a unique "lock-and-key" mechanism, which is distinct from most other 14-3-3 interactions (30).

SIK2 and SIK3 are implicated in sequestering CRTC2 in the cytoplasm in the basal state: siRNA knockdown or inhibition of these SIKs increased PTH-induced *Tnfsf11* mRNA abundance and CRTC2 nuclear translocation. Putative activation of SIKs reduced PTH-treated *Tnfsf11*, suggesting this may be a mechanism to ameliorate long-term negative effects of the PTH pathway. PP1, 2, 4, and 5 knockdown significantly reduced PTH-treated *Tnfsf11* expression and CRTC2 translocation, showing their activity is crucial for dephosphorylation of CRTC2 with subsequent nuclear shuttling. Co-immunoprecipitations of SIKs and PPs with CRTC2 corroborate proposed interactions with each other, and changes in physical interactions were shown between basal and PTH-treated states. Calcineurin (PP3) knockdown caused significant increases in basal and PTH-treated *Tnfsf11*, suggesting it may be a negative regulator of this cascade in the osteoblast. Finally, PP6 and PP7 do not appear to be involved.

These findings highlight the functional diversity of protein phosphatases within cellular signaling networks. Although similar results were found by knocking down PP1, 2, 4, and 5, they have unique regulatory mechanisms and substrate specificities (26, 31). PP1 and PP2 interact with a wide array of regulatory subunits, guiding them to specific substrates and cellular locations (32, 33). PP2's versatility is highlighted by its ability to form diverse heterotrimeric structures, significantly influencing its substrate specificity and localization. On the other hand, PP4 and PP5 are more direct with their action, with PP5 notably featuring a TPR (tetratricopeptide repeat) domain that aids in its specific regulatory actions (34). PP3's activity is modulated by calcium ions and calmodulin, linking it directly to calcium signaling pathways (35). This unique mode of activation allows PP3 to play a pivotal role in calcium-dependent cellular responses, and its activity may have differing downstream effects, or by interacting with other phosphatases/factors to increase their activity.

Although the data presented offers a wealth of knowledge regarding the role of CRTC proteins, SIKs, and PPs on *Tnfsf11* transcription, further work should explore the opportunity for additional mechanisms such as structural differences in the CRTC, SIK, and PP isoforms. The activity of PPs is nuanced and requires additional examination of their characteristics to provide further insight into the results we found. Additionally, our data found substantial compensation and cross-regulation in our knockdowns. For every siRNA knockdown with qRT-PCR we assessed fold change of >40 genes (all *Crtc*s, *Sik*s, PP subunits, bZIPs, and additional genes of interest such as *Vdr*, Wnt family members (36) *etc.*) from harvested RNA and almost all knockdowns led to cross-regulation (data not shown) similar to that we showed with *Crtc2*/*3*. To improve our understanding of these interactions, future studies could incorporate rescue experiments, where exogenous CRTC3 is introduced back into the cells after CRTC2 and/or CRTC3 knockdown to assess its potential to rescue the observed phenotype. This would offer more definitive insight into the specific role and importance of CRTC3 in this context. Similar rescue experiments could be designed to explore the potential compensatory interactions among other factors studied. Understanding this dynamic is crucial, as it can affect the interpretation of results and the design of future experiments.

A limitation of the study is that it was only conducted in primary calvarial osteoblasts, which are not pure cells. However, we have found that these cells are the best model for examination of PTH regulation of *Tnfsf11* transcription since they show such a great stimulation (15–50-fold). We have tried a number of experiments with UMR 106-01 cells (not shown) and found that they only exhibit about 2-3-fold PTH stimulation of *Tnfsf11*. In fact, immunofluorescence studies with these cells have revealed that they do not show nuclear translocation of CRTC2 or 3, which could account for the low PTH stimulation of *Tnfsf11*. We have found in the past that the MC3T3-E1 cells are poorly responsive to PTH and, thus, a poor model for PTH action. We have tested all the human osteosarcoma lines available, and only SaOS-2 cells show any PTH responsiveness, and even that is low. The primary calvarial osteoblasts are also normal cells, not a cancer cell line, nor immortalized or transformed, unlike MC3T3-E1 cells or all the osteocyte lines. A limitation of the primary calvarial cell model is our inability to detect changes in RANKL at the protein level (data not shown). However, RANKL is a complicated protein, a trimeric transmembrane protein, possibly already complexed with osteoprotegerin intracellularly, which can be cleaved by several metalloproteases or ADAMs, releasing sRANKL into the medium. There, it can signal to cells bearing RANK, or the transmembrane form can even signal back to osteoblasts when bound to RANK (37) through reverse signaling. We consider that our inability to observe similar stimulation of the protein as the mRNA reflects this complicated pathway, which needs further investigation outside of the scope of this manuscript.

Immunofluorescence data showed 40% of basal osteoblasts have CRTC2 in their nucleus, and >90% of PTH-treated osteoblasts have CRTC2 in their nucleus. This is the expected trend based on our model, but the 40% in the basal state seems higher than expected for how little *Tnfsf11* mRNA was found compared to PTH-treated samples. While CRTC2 activity on *Tnfsf11* transcription is confirmed following PTH stimulation, it could also have basal activity that is lesser known or understood. This could result in a proportion of CRTC2 being found in the nucleus under basal conditions. The osteoblasts could be responding to unidentified stimuli in their environment that cause CRTC2 to translocate to the nucleus, even under basal conditions. There might be a threshold level of nuclear CRTC2 needed to stimulate significant *Tnfsf11* production. It is also likely that potential transcription factors that bind to the CRTCs, such as CREB family members, are tightly regulated by phosphorylation by PKA or by other stimuli. Required interactions to initiate *Tnfsf11* transcription may still be rare in the basal state despite a high amount of CRTC2 in the nucleus.

Similarly, we found that siRNA knockdown of SIK2/3 led to increased basal and PTH-treated *Tnfsf11* mRNA compared to control osteoblasts but since control cells already have close to 100% CRTC2 in their nucleus after PTH-treatment the increased *Tnfsf11* cannot be due to increased CRTC2 translocation alone. This is likely also to require PKA phosphorylation of transcription factors such as CREB family members.

Preliminary data of ours (not shown) have indicated that CREB1 is not the major CREB family member responsible for PTH stimulation of *Tnfsf11* transcription in osteoblasts, but instead a member (or members) of the CREM isoforms appears to be more important. This work is ongoing, since CREM has 29 isoforms and is a complex gene with both stimulatory and inhibitory family members, as well as feedback regulation (38).

In conclusion, this study provided significant insights into the intricate mechanisms of PTH-induced *Tnfsf11* transcription, shedding light on the roles of several previously unstudied elements that appear to be critical factors from PTH-treatment → CRTC translocation → *Tnfsf11* transcription (Fig. 8). The results offer novel potential therapeutic strategies for the treatment of osteoporosis and contribute to our understanding of bone maintenance/remodeling.

## Experimental procedures

### Peptides and chemicals

Rat parathyroid hormone (PTH 1–34) was purchased from Bachem, which was confirmed and analyzed for purity and degradation by the NYU Grossman School of Medicine Mass Spectrometry Core Facility. PTH (1–34) was dissolved in 10 mM acetic acid and snap frozen for future experiments. RIPA buffer, ascorbic acid, and AICAR (5-aminoimidazole-4-carboxamide ribonucleoside) were purchased from Sigma. Collagenase A was purchased from Worthington Biochemical Corporation. SIK inhibitors (YLK-06-061, HG-9-91-01, ARN-3236) were obtained from Selleckchem.

### Cell culture

Primary mouse calvarial osteoblasts were harvested from C57Bl/6J wild-type mice aged 2 to 3 days postnatal. The procedures and studies were approved by the NYU Grossman School of Medicine IACUC. Mice were euthanized with ketamine (0.25 mg/pup) and xylazine (0.025 mg/pup). Calvariae were digested in 10 mg/ml collagenase A at 37 ^o^C by five sequential digestions, and cells from digests 3 to 5 were collected and plated at a density of 6.4 x 10^3^ cells/cm^2^ in αMEM supplemented with 10% FBS, 100 units/ml penicillin, 100 ug/ml streptomycin, and 0.25 μg/ml of amphotericin b. After reaching confluence, which typically occurred within 5 days of cells being plated, if osteogenic differentiation was necessary the medium was replaced and supplemented with 50 ug/ml ascorbic acid and cells allowed to differentiate for additional time based on the protocol chosen: (1) undifferentiated osteoblasts: 5 days total in αMEM, (2) mature osteoblasts: 5 days in αMEM + 2 days in osteoblast differentiation medium, and (3) late-stage mineralized osteoblasts: 5 days in αMEM + 7 days in osteoblast differentiation media. For additional information on how these protocols were determined see (Fig. S4). Prior to harvest, osteoblasts were treated with 10 nM PTH (1–34) for 4 h for RNA/protein isolations, 45 min for immunofluorescence, or 15 min for co-immunoprecipitations.

### siRNA knockdowns

Lipofectamine RNAiMAX Transfection Reagent (Invitrogen) was added 48 h prior to cell treatment with PTH (1–34) and cell harvest or fixation. Duplex sequences of the siRNAs used are shown in the (Table S1). Lipofectamine RNAiMAX was diluted in 150 μl of Opti-MEM Medium. siRNAs were simultaneously diluted in Opti-MEM Medium and both solutions were incubated separately for 15 min. Diluted siRNAs were then added to diluted Lipofectamine RNAiMAX Reagent (1:1 ratio) and were then incubated for an additional 5 min prior to adding siRNA-lipid complex to plated osteoblasts in 6-well plates (1 ml medium/well). Confirmation of knockdowns was determined for mRNA abundance by qRT-PCR for mRNA and protein levels by Western blotting (Figs. S5 and S6).

### qRT-PCR

Total RNA was extracted using Trizol (Sigma). Complementary DNA (cDNA) was synthesized from one ug of total RNA using TaqMan reverse transcription kit (Applied Biosystems) with hexamer primers following manufacturer’s protocol. Gene expression levels were measured using SYBR Green PCR Reagents (Applied Biosystems). Mouse primers used for qRT-PCR are shown in (Table S2). The quantity of mRNA was calculated by normalizing the threshold cycle value (Ct) of specific genes to the Ct of the housekeeping gene *Ribosomal protein* L*13a* (*Rpl13a*). All values shown in figures represent individual experiments with each data point representing the average value calculated from three technical replicates of three wells.

### Western blotting

Total cell lysates were prepared in a 1× RIPA buffer (Bio Basic, Canada), which included phosphatase and protease inhibitors. Proteins were resolved on 12% SDS–polyacrylamide gels. They were then electrophoretically transferred to a PVDF membrane (Bio-Rad). The membrane was blocked with Tris-buffered saline containing 0.1% Tween 20 (TBS-T) and 5% BSA, followed by incubation with primary antibody overnight at 4 °C. Antibodies used can be found in (Table S3). The membrane was then reacted with Thermo Clean blot secondary antibody (HRP conjugated) for 1 h at 4 °C. The membrane was washed three times with TBS-T and was developed using an ECL kit and imaged using Bio-Rad software.

### Quantitative immunofluorescence

Cells in culture chambers were fixed with 3.7% paraformaldehyde, washed and then permeabilized with 0.002% Triton X-100 for 30 min, followed by blocking with 1% BSA in 1× PBS for 1 h. Cells were incubated with 1:250 dilution of primary antibodies (see Table S3) for 1 h, followed by washes and incubation with 1:450 dilution of Alexa Fluor 488-conjugated secondary antibodies. Cells were incubated with 1:10,000 DAPI (Life Technologies) for nuclear staining and mounted using Fluoromount-G fluorescence mounting medium (Invitrogen) and images were acquired using a Nikon Eclipse E600 microscope. 20 images of each stained well (three independent wells/final value) were acquired in a systematic manner at 40x magnification by a single blinded investigator with a second blinded investigator checking calculations *via* a random sampling of 20 images from the stained slide. For CRTC2 immunostaining a cell ratio was calculated manually: positively stained nuclear cells/total cells. Once positive cells and total cells were counted for each image, the results were summed for each sample to give total positive cells/total cells, and the presented data are shown as a percentage.

Confocal imaging was performed using a Zeiss LSM 900 equipped with solid-state diode lasers. DAPI was excited using a 405 nm laser, and emission was collected between 410 to 470 nm and Alexa Fluor 488 was excited using a 488 nm laser, and emission was collected between 505 to 617 nm. Images were acquired using a 20× objective lens. Emission channels were carefully separated to minimize spectral overlap and fluorescence crosstalk.

### Co-immunoprecipitation

Cells were washed with 1× PBS and lysed in lysis buffer containing 0.5% Nonidet P-40, 50 mm Tris (pH 8.0), 150 mm NaCl, 1 mM EDTA, and Halt Protease Inhibitor Cocktail (Thermo Scientific). Cells were scraped and incubated at 4 °C for 20 min. Cellular debris was pelleted by centrifugation. The supernatant was precleared by the addition of either 1 μg of rabbit IgG or 1 μg of mouse IgG and 20 μl of protein AG-agarose, incubating for 1 h at 4 °C, and centrifuging to pellet the agarose. Equal amounts of lysates were mixed with either 1 μg of monoclonal or 1 μg of polyclonal antibodies and 20 μl of protein AG-agarose. The samples were incubated overnight at 4 °C and washed three times with lysis buffer. Next, the samples were resuspended in SDS sample buffer + lysis buffer and boiled for 5 min. Both precleared lysates and immunoprecipitated samples were resolved by 12% SDS-polyacrylamide gels. The proteins were transferred electrophoretically to polyvinylidene difluoride membranes. The membranes were immunoblotted with either monoclonal or polyclonal antibodies.

### Statistics

Each individual data point shown in the figures represents a separate experiment in which the value was averaged from technical triplicates of three wells. Standard deviation is calculated using the averaged value over three experiments. Therefore, results are shown as mean ± SD where n = 3. Statistical differences were calculated using GraphPad Prism (v9). Normality was tested using Shapiro-Wilk test for normality and, depending on the analyses conducted, one-way and two-way ANOVAs with *post hoc* Tukey tests were used to determine significance. A three-way ANOVA was used for interaction *p* values. This was followed by two-way ANOVAs with *post hoc* Tukey tests for each differentiation status in Figure 1. *p* < 0.05 was considered significant comparing groups and the *p* values are represented by ∗*p* < 0.05, ∗∗*p* < 0.01, ∗∗∗*p* < 0.001.

## Data availability

The corresponding author can provide the datasets created and/or analyzed during the current work upon reasonable request.

## Supporting information

This article contains Supporting Information (39, 40, 41, 42, 43, 44).

## Conflict of interest

The authors declare that they have no conflicts of interest with the contents of this article.
