# Supplementary material for: PTH stimulation of Rankl transcription is regulated by SIK2 and 3 and mediated by CRTC2 and 3 through action of protein phosphatases 1, 2, 4, and 5
Source: J Biol Chem. 2025 Jul 1;301(8):110434. doi: 10.1016/j.jbc.2025.110434 (PMC12359225; doi:10.1016/j.jbc.2025.110434)
Supplement: Supporting information figures [file mmc1.docx]

PTH stimulation of Rankl transcription is regulated by SIK2 and 3 and mediated by CRTC2 and 3 through action of protein phosphatases 1, 2, 4, and 5

Michael J Mosca, Zhiming He, Nagarajan Selvamurugan, Jobin Joseph, Whitney Petrosky, Carole Le Henaff, and Nicola C. Partridge

**Supporting Information:**

**Figure S1: Expression of *Rpl13a* throughout differentiation stages with variable lipofectamine and siRNA concentrations**.

**Figure S2: Expression of *Crtc1, Crtc2,* and *Crtc3* mRNAs throughout differentiation stages with variable lipofectamine and siRNA concentrations.**

**Figure S3: Relative expression of *Rpl13a* mRNA with siRNA knockdowns/SIK inhibitors and determination if siRNA concentration alone affects *Tnfsf11* mRNA** **and PTH stimulation.**

**Figure S4: Expression of osteogenic genes throughout differentiation protocols.**

**Figure S5: Confirmation of siRNA knockdowns used by qRT-PCR**.

**Figure S6: Confirmation by Western blotting of siRNA knockdowns used.**


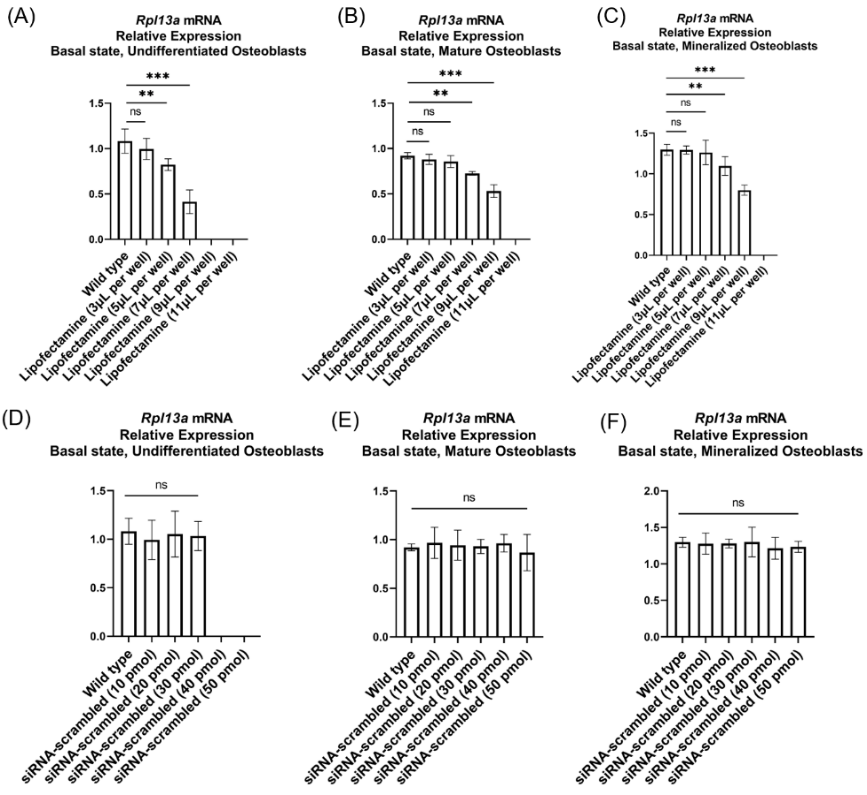


**Figure S1: Expression of *Rpl13a* throughout differentiation stages with variable lipofectamine and siRNA concentrations**. Here we determined the optimal amount of lipofectamine reagent and siRNAs to be used with the goal of maximally knocking down the CRTCs without damaging the cells’ basic functions or leading to cell death. Lipofectamine® RNAiMAX manufacturer’s protocol suggests 7 μL and 30 pmol of siRNA be used for each well when using 6 well plates (1 ml medium/well) but that these values may require optimization, especially for primary cells. Primary calvarial osteoblasts were plated according to three differentiation protocols: 1) undifferentiated (5 days in αMEM media), 2) mature (5 days to reach confluence + 2 days in osteogenic differentiation media), and 3) mineralized (5 days to reach confluence + 7 days in osteogenic differentiation media). For all time points lipofectamine alone or scrambled siRNA alone was added 48 h prior to harvest at varying amounts. If cells survived, they were harvested for RNA and cDNA synthesized for qRT-PCR analyses of *Rpl13a* mRNA. Blank values on graphs represent protocols that led to cell death. Data represent relative expression of *Rpl13a* mRNA, * p < 0.05, ** p <0.01, *** p < 0.001. ANOVA followed by post hoc Tukey tests used. We found that 3 μL of lipofectamine did not significantly affect *Rpl13a* mRNA at any differentiation stage, 5 μL only significantly affected *Rpl13a* in undifferentiated cells by a small amount, 7 μL significantly decreased *Rpl13a* in all three stages but did so by a large amount in undifferentiated cells, >9 μL lead to cell death in undifferentiated cells with significantly decreasing *Rpl13a* in mature and mineralized cells, and 11 μL of lipofectamine lead to cell death in all three stages of differentiation. Scrambled siRNA alone had no significant effect on *Rpl13a* mRNA at any concentration up to 50 pmol in mature and mineralized osteoblasts but greater than 40 pmol and above led to cell death in undifferentiated cells.


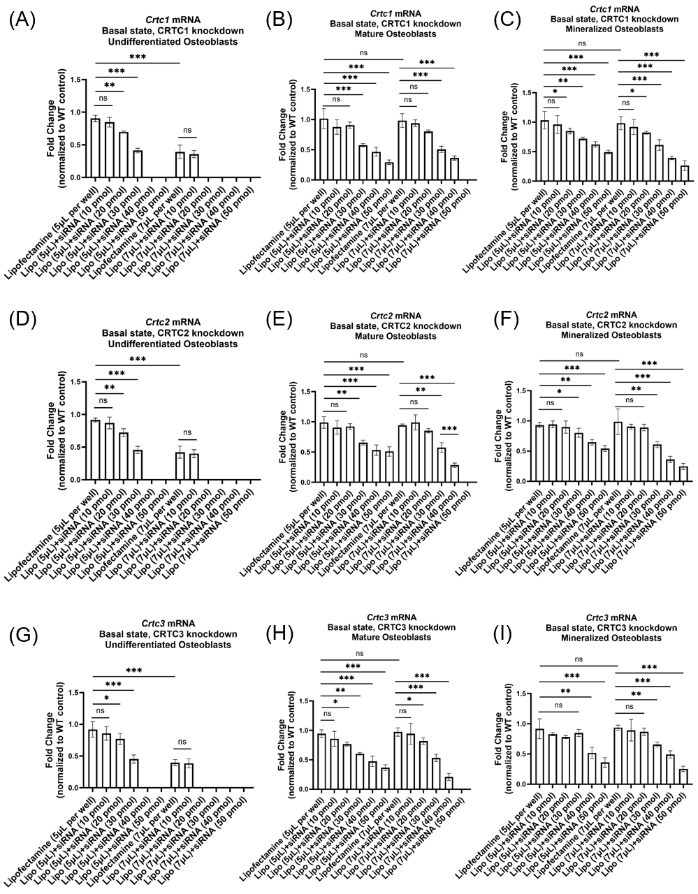
**Figure S2: Expression of *Crtc1, Crtc2,* and *Crtc3* mRNAs throughout differentiation stages with variable lipofectamine and siRNA concentrations**. We further examined the effects of combining several concentrations of siRNAs for *Crtc1*, *Crtc2*, and *Crtc3* (ranging from 10 pmol to 50 pmol, in 6 well plates with 1 ml medium/well) with both 5 μL and 7 μL of lipofectamine. The goal was to determine a consistent dosage to be used in each differentiation protocol that would not substantially affect cell function but that would also result in significant reduction in targeted gene expression. Ideally, we would not have to increase lipofectamine or siRNA concentration as cells differentiate. Primary calvarial osteoblasts were plated according to three differentiation protocols: 1) undifferentiated (5 days in αMEM media), 2) mature (5 days to reach confluence + 2 days in osteogenic differentiation media), and 3) mineralized (5 days to reach confluence + 7 days in osteogenic differentiation media). For all time points lipofectamine and siRNAs were added 48 h prior to harvest at varying amounts. If cells survived, they were harvested for RNA and cDNA synthesized for qRT-PCR analyses of *Crtc* mRNAs at each time point. Blank values on graphs represent protocols that led to cell death. Data represent fold change normalized to wild type control samples at the same differentiation stage, * p < 0.05, ** p <0.01, *** p < 0.001. One-way ANOVA with Tukey’s tests used.

Data were analogous among *Crtc1, Crtc2,* and *Crtc3* mRNAs for each dosage of lipofectamine + siRNAs. For example, in undifferentiated osteoblasts a significant reduction in all *Crtc* mRNAs on the order of ~50% occurred when increasing lipofectamine dosage from 5 μL to 7 μL and any concentration of siRNA above 10 pmol with 7 μL of lipofectamine caused cell death. Additionally for undifferentiated cells, 5 μL of lipofectamine with 30 pmol of siRNA led to the greatest reduction in mRNA expression regardless of CRTC with any additional siRNA leading to cell death. Due to this, if we wanted to have a consistent protocol throughout all three differentiation stages 5 μL of lipofectamine with 30 pmol of siRNA would be the largest possible amount to utilize. In order to reach ~50% reduction in *Crtc1, 2, & 3* mRNAs in mature and mineralized osteoblasts keeping lipofectamine dose at 5 μL, an increase to 40 pmol would be required. Luckily this did not affect *Rpl13a* mRNA and did not appear to affect the cells in any other respect other than increasing the effectiveness of the knockdowns. These data led us to determine that the undifferentiated protocol would use 5 μL + 30 pmol of siRNAs and the mature and mineralized protocols would use 5 μL + 40 pmol of siRNAs. Further knockdown could be achieved in mature and mineralized osteoblasts by increasing lipofectamine dosage but would add additional variability when comparing several differentiation stages.

**
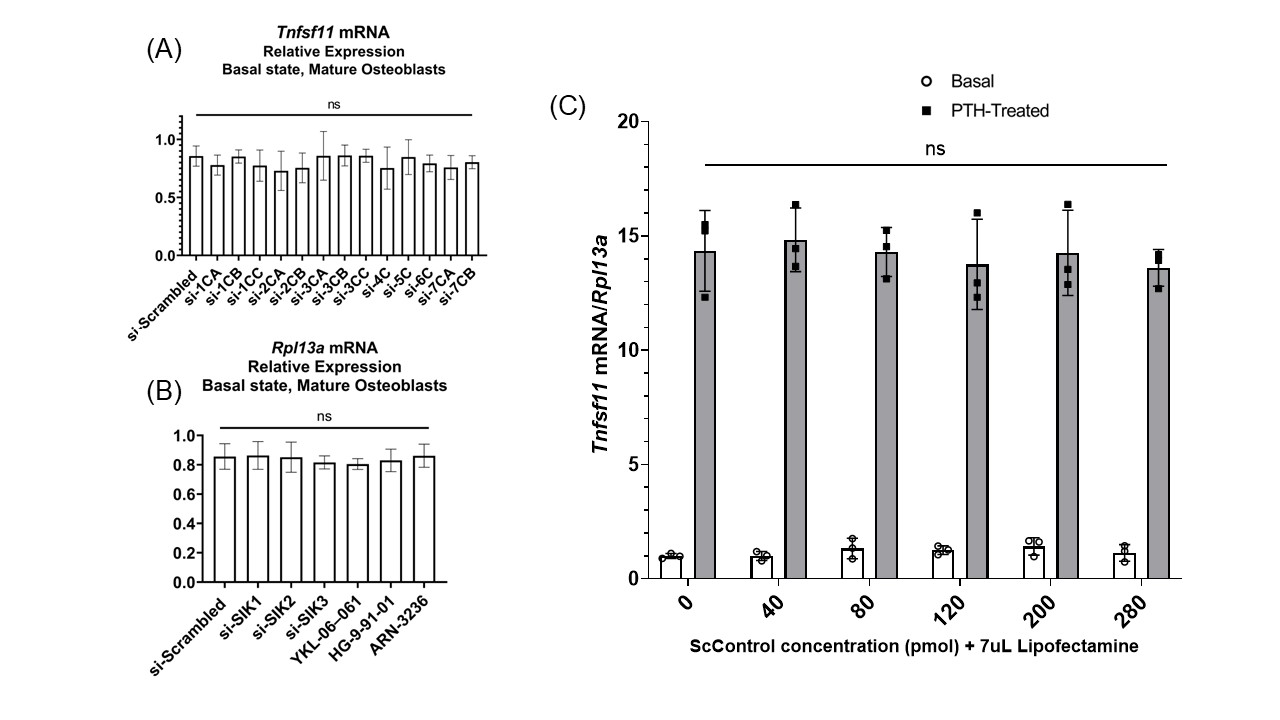
**

**Figure S3: Relative expression of *Rpl13a* mRNA with siRNA knockdowns/SIK inhibitors and determination if siRNA concentration alone affects *Tnfsf11* mRNA** **and PTH stimulation.** Before data from siRNA knockdowns could be assessed, we confirmed that the protocols would provide sufficient knockdowns of each factor of interest and that baseline cell function would not be significantly altered. Protocol previously determined used a lipofectamine dose of 7 µL and 40 pmol of siRNAs in mature cells in 6 well plates (1 ml medium/well) and we determined if siRNAs produced adequate knockdowns by qRT-PCR and Western blotting using this protocol (Figures S5, S6). siRNA knockdowns of each SIK and each catalytic subunit of all PPPs produced significant reduction by a minimum of 50% knockdown in both mRNA abundance and protein levels. From these knockdowns we also determined *Rpl13a* mRNA relative expression to WT controls (S3, A). We also tested the effect our SIK inhibitors (YKL-06-061, HG-9-91-01, & ARN-3236) had on *Rpl13a* (S3, B). We found no significant difference in *Rpl13a* mRNA in any knockdowns or treatment with SIK inhibitors compared with controls. Since variable siRNA concentrations would be used for combination knockdowns later, we confirmed that additional scRNA concentrations did not alter *Tnfsf11* mRNA or PTH stimulation as long as lipofectamine dose remained constant (Figure S3, C).

**
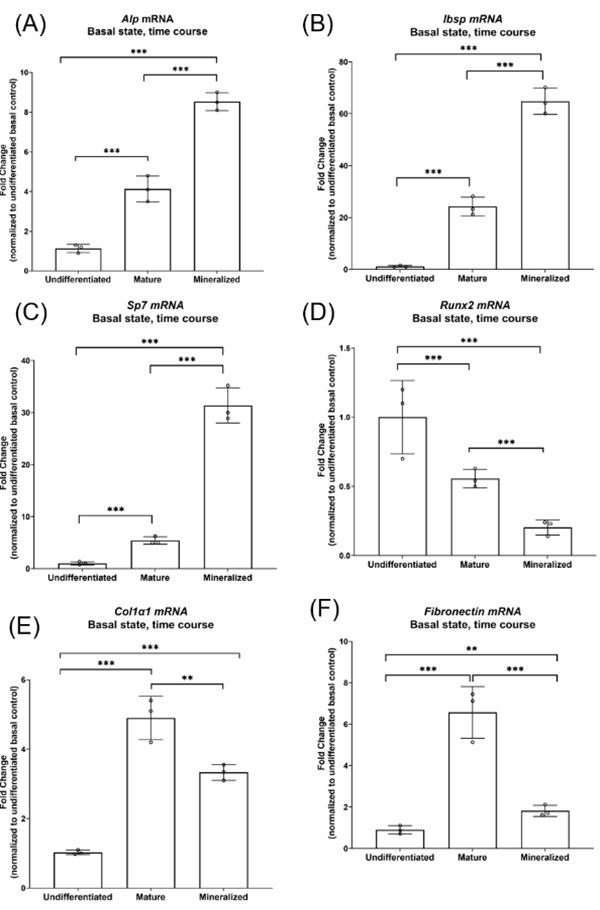
Figure S4: Expression of osteogenic genes throughout differentiation protocols.** Before siRNA knockdowns could begin, we first determined the protocols that would be used to consistently differentiate the calvarial osteoblasts to distinguishable stages of osteogenic maturation. Protocol from previous work in our laboratory using the primary mouse calvarial osteoblast in vitro model changed the cells from αMEM to osteogenic (αMEM + ascorbate) after cells reached confluence, which typically occurred within 5 days of plating. After confluence was reached, osteogenic media was added for additional days until cell harvest. Our study proposed to create protocols to differentiate our osteoblasts into three distinct stages: 1) undifferentiated osteoblasts, 2) mature osteoblasts, and 3) late stage mineralized osteoblasts. Based on manufacturer’s protocol of Lipofectamine® RNAiMAX Reagent, siRNA transfection was optimal after 48 h of incubation, so our starting point for undifferentiated osteoblasts was a minimum of 5 days post plating. Importantly, cells which were subjected to the undifferentiated protocol would have simple αMEM supplementation with no osteogenic media added at any point. The protocol for mature osteoblasts was 5 days to reach confluence + 2 days in osteogenic media for a total of 7 days prior to treatment and harvest. For late-stage osteoblasts we wanted the cells to be mineralized. We tried several time points and settled on 5 days to reach confluence + 7 days in osteogenic media for a total of 12 days. This time was adequate to produce osteoblasts with significantly different expression profiles compared to mature and undifferentiated cells.

Primary calvarial osteoblasts were harvested from C57BL/6J mice aged 2-3 days postnatal; calvariae were digested and cells were plated according to three differentiation protocols: 1) undifferentiated (5 days in αMEM media), 2) mature (5 days to reach confluence + 2 days in osteogenic differentiation media), and 3) mineralized (5 days to reach confluence + 7 days in osteogenic differentiation media). Cells were then harvested for RNA and cDNA synthesized for qRT-PCR analyses of (A) *Alp* mRNA, (B) *Ibsp* mRNA, (C) *Sp7* mRNA, (D) *Runx2* mRNA, (E) *Col1α1* mRNA, and (F) *Fibronectin* mRNA. Data represent fold change normalized to undifferentiated osteoblasts, * p < 0.05, ** p <0.01, *** p < 0.001. ANOVA followed by post hoc Tukey tests showed *Alp* mRNA, *Ibsp* mRNA, and *Sp7* mRNA were all increased significantly from undifferentiated 🡪 mature 🡪 mineralized cells (S1, A-C). *Runx2* mRNA decreased significantly from undifferentiated 🡪 mature 🡪 mineralized cells (S4, D). *Col1α1* and *Fibronectin* mRNAs were highest in mature osteoblasts, less in both undifferentiated and mineralized cells (S4, E-F). These data follow expected expression patterns between the different stages of osteogenic differentiation.^39-44^

**
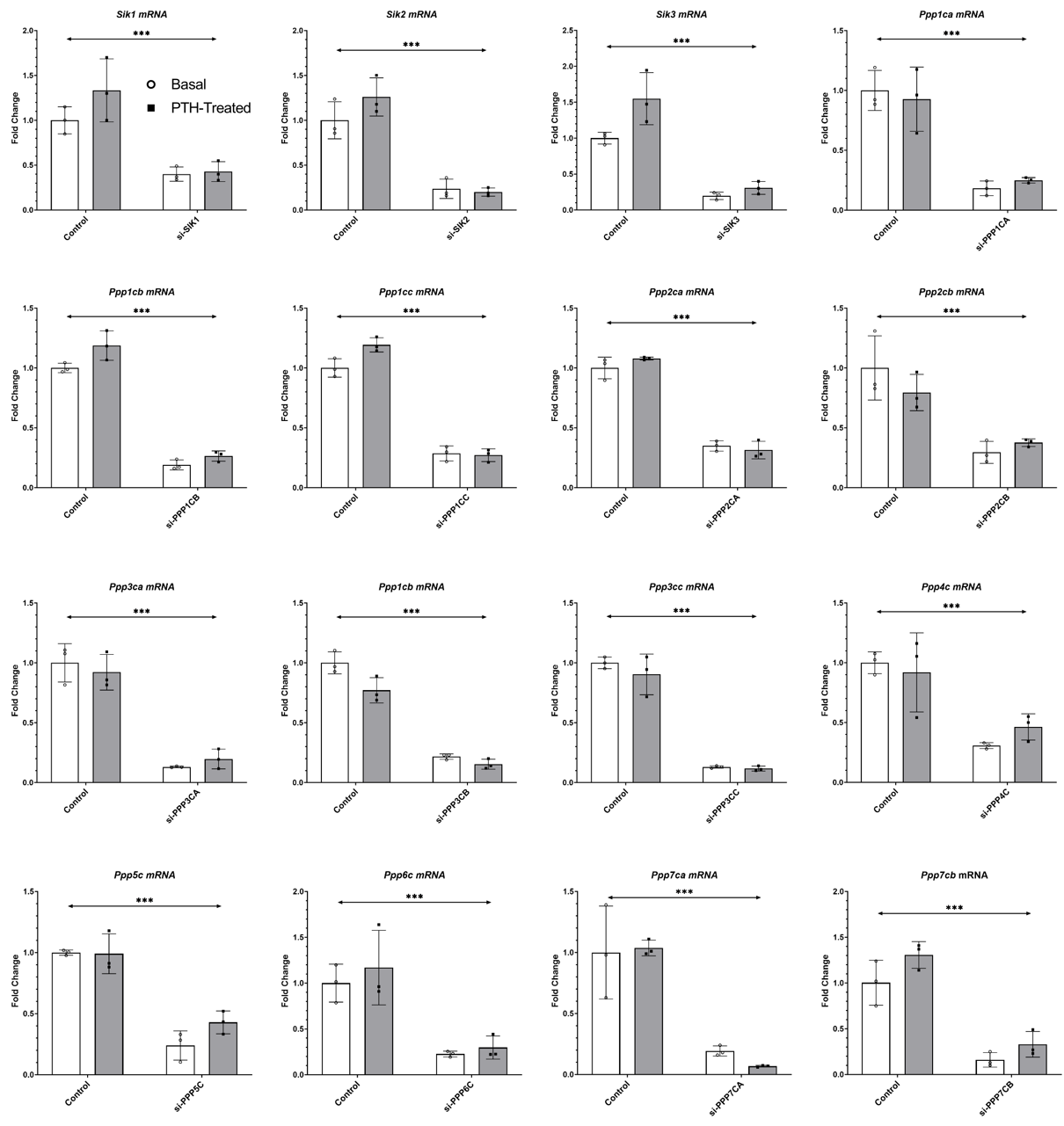
**

**Figure S5: Confirmation of siRNA knockdowns used by qRT-PCR**. Primary calvarial osteoblasts were plated in 6-well plates for 5 days to reach confluence + 2 additional days in osteogenic differentiation medium. siRNAs (40 nM) were added for the last 48 h prior to 10 nM PTH (1-34) treatment for 4 h and harvested for RNA. cDNA was synthesized for qRT-PCR analyses of *Siks1-3* and *Pps 1-7* mRNA. Data represent fold change normalized to siRNA scrambled basal control, * p < 0.05, ** p <0.01, *** p < 0.001. One-way ANOVA with post hoc Tukey tests used.


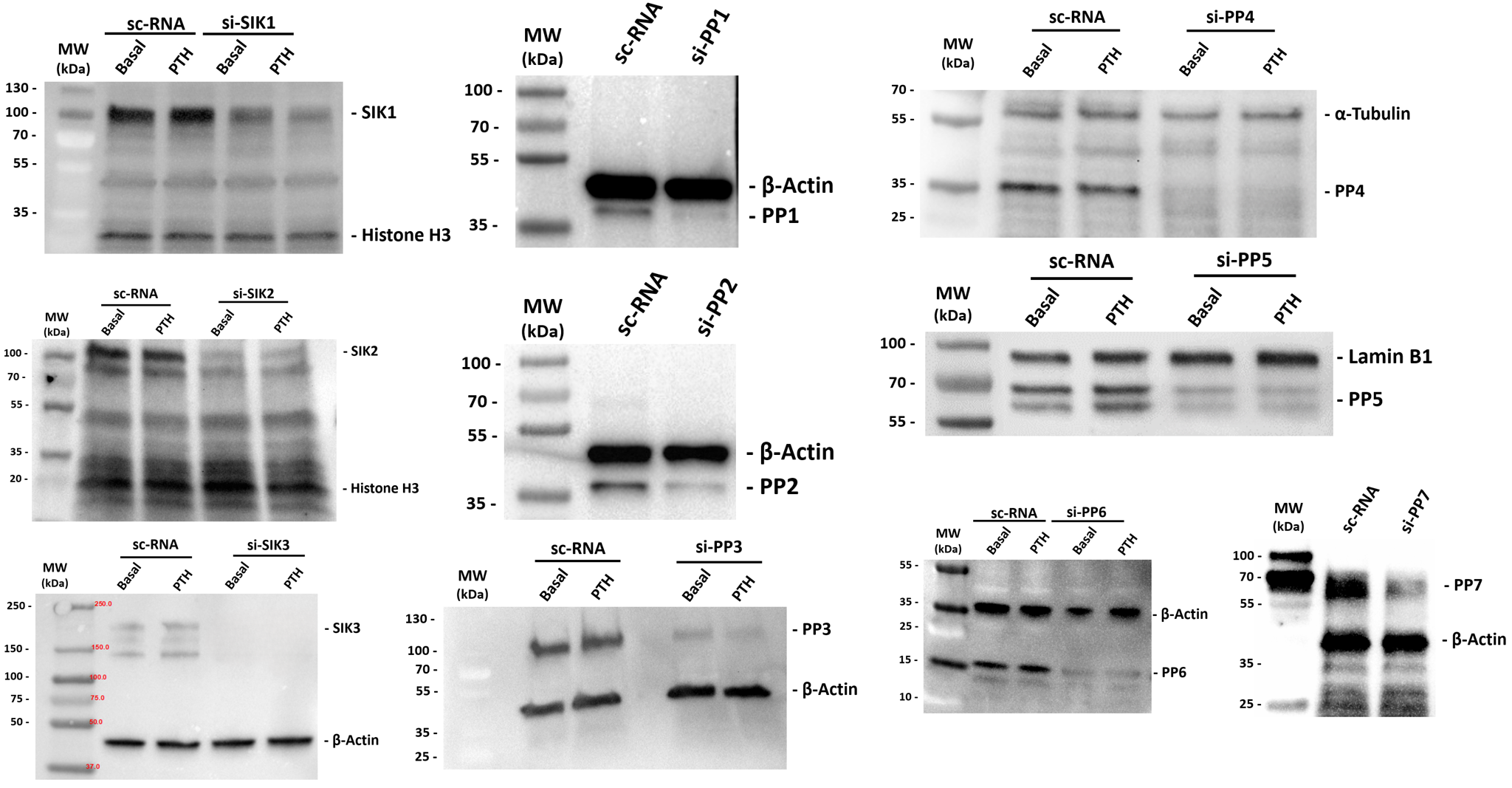


**Figure S6: Confirmation by Western blotting of siRNA knockdowns used**. Primary calvarial osteoblasts were plated in 100 mm dishes for 5 days to reach confluence + 2 additional days in osteogenic differentiation media. siRNAs were added 48 h prior to harvest. Proteins were isolated and Western blots conducted to determine if levels were reduced.
