# Supplementary material for: PTH stimulation of Rankl transcription is regulated by SIK2 and 3 and mediated by CRTC2 and 3 through action of protein phosphatases 1, 2, 4, and 5
Source: J Biol Chem. 2025 Jul 1;301(8):110434. doi: 10.1016/j.jbc.2025.110434 (PMC12359225; doi:10.1016/j.jbc.2025.110434)
Supplement: Table S1 [file mmc2.docx]

| **Table S1: siRNA sequences used for in vitro transfection** | |
| --- | --- |
| **DsiRNA (mm.Ri)** | **Duplex Sequence (5’ 🡪 3’)** |
| Scrambled negative control | rCrUrUrCrCrUrCrUrCrUrCrUrUrUrUrCrUrCrUrCrCrCrUrUrGrUGA  rUrCrArCrArArGrGrGrArGrArGrGrArArArGrArGrArGrGrArArGrGrA |
| Crtc1 | rArGrC rArUrU rGrCrU rUrUrG UrCrC rArArG  rCrArG rGrUrG rArCrU rUrGrG rArCrA rArArA |
| Crtc2 | rCrCrC rUrUrG rGrArU rArCrC rArGrU rArArA  rUrArG rGrCrA rArUrU rUrArC rUrGrG rUrArU |
| Crtc3 | rGrArC rArGrA rArGrA rUrArC rArArA rCrArG  rCrArG rGrUrU rGrCrU rGrUrU rUrGrU rArUrC |
| Sik1 | rArArG rArArC rUrUrU rCrUrA rGrArC rGrUrG  rCrUrU rGrUrU rUrCrA rCrGrU rCrUrA rGrArA |
| Sik2 | rCrArU rGrGrA rArGrC rUrUrC rCrArA rGrCrC  rGrArG rArArA rArGrG rCrUrU rGrArA rArGrA |
| Sik3 | rUrUrC rUrGrG rArCrG rCrCrA rArUrC rUrGrA  rUrUrG rArUrA rUrUrC rArGrA rUrUrG rGrCrG |
| Ppp1ca | rCrArA rGrCrA rUrGrA rUrUrU rGrGrA rCrCrU  rGrCrA rGrArU rGrArG rGrUrC rCrArA rArUrC |
| Ppp1cb | rArGrU rArArA rUrUrU rCrUrG rArArU rCrGrU  rArArU rCrArU rGrArC rGrArU rUrCrA rGrArA |
| Ppp1cc | rCrGrU rGrArG rUrCrU rCrUrA rCrArU rGrUrA  rUrGrA rCrArA rUrUrA rCrArU rGrUrA rGrArG |
| Ppp2ca | rGrArC rGrCrG rArCrA rUrUrG rUrUrG rGrUrU  rGrArU rUrCrU rUrGrA rCrCrA rArCrA rArUrG |
| Ppp2cb | rArUrG rGrArA rUrUrA rGrArU rGrArC rArCrU  rArUrU rUrUrA rArArG rUrGrU rCrArU rCrUrA |
| Ppp3ca | rGrGrA rUrUrC rArGrU rUrUrA rCrArC rUrArU  rUrUrU rCrArU rGrArU rArGrU rGrUrA rArArC |
| Ppp3cb | rGrArC rCrArA rGrGrU rGrArU rGrArU rArArA  rUrArU rGrArC rArUrU rUrArU rCrArU rCrArC |
| Ppp3cc | rCrArA rCrArG rUrUrU rArUrU rArUrC rArArU  rUrCrU rGrArU rUrArU rUrGrA rUrArA rUrArA |
| PP4c | rGrCrU rArUrC rCrUrG rArCrA rGrArA rUrCrA  rArUrC rArArA rGrUrG rArUrU rCrUrG rUrCrA |
| PP5c | rGrCrG rArUrC rArArG rUrUrC rUrArC rArGrU  rUrGrG rCrCrU rGrArC rUrGrU rArGrA rArCrU |
| PP6c | rArArG rArUrG rUrCrA rArUrA rCrGrA rGrArG  rUrUrU rGrGrU rUrCrU rCrUrC rGrUrA rUrUrG |
| Ppp7ca | rCrArG rUrGrC rUrUrU rCrArA rGrArU rArCrU  rUrUrC rCrUrU rUrArG rUrArU rCrUrU rGrArA |
| Ppp7cb | rGrArA rUrCrU rUrCrU rGrUrA rCrGrA rGrArC  rUrUrU rUrCrU rGrGrU rCrUrC rGrUrA rCrArG |
