# Supplementary material for: PTH stimulation of Rankl transcription is regulated by SIK2 and 3 and mediated by CRTC2 and 3 through action of protein phosphatases 1, 2, 4, and 5
Source: J Biol Chem. 2025 Jul 1;301(8):110434. doi: 10.1016/j.jbc.2025.110434 (PMC12359225; doi:10.1016/j.jbc.2025.110434)
Supplement: Table S2 [file mmc3.docx]

| **Table S2: Mouse primer sequences used for qRT-PCR** | | |
| --- | --- | --- |
| **Gene** | **Forward** | **Reverse** |
| Rpl13a | CTGCTCTCAAGGTTGTTCGGCT | CCTTCCGTTTCTCCTCCAGAGT |
| Rankl | GCTCCGAGCTGGTGAAGAAA | CCCCAAAGTACGTCGCATCT |
| Crtc 1 | TGCCCAACGTGAACCAGATT | CCCATGATGTCGTGTGGTCC |
| Crtc 2 | ATGAACCCTAACCCCCAAGAC | CGTTCTCCTCAATAGCAGGGA |
| Crtc 3 | TGACTCACCTGGGGATAAGAAC | GTGGCACTTGAGGGACGAG |
| Sik 1 | TCATGTCGGAGTTCAGTGCG | ACCTGCGTTTTGGTGACTCG |
| Sik 2 | CTGCTGGCAACATGGTGTG | GGGAGAGTTGGTCCATCAAAAG |
| Sik 3 | GCCATCCACACATCATCAGAC | CCAAGTGGTCAAATATCTCCCC |
| Ppp1ca | ATGTCCGACAGCGAGAAGC | ACAGCCGTAGAAGGTCATAGT |
| Ppp1cb | GATGTCGTCCAGGAAAGATTGT | TCAGTGGTGCTTCCAATTCCA |
| Ppp1cc | GAGAACGAGATCCGAGGACTC | CGTATTCAAACAGACGGAGCAA |
| Ppp2ca | AAGGTTCGTTACCGAGAGCG | GGTGACAGACCACCGTGTAG |
| Ppp2cb | CTGAACGAGAACCAAGTGCG | GGTCACATGAGGCTCTCCAC |
| Ppp3ca | GTGAAAGCCGTTCCATTTCCA | GAATCGAAGCACCCTCTGTTATT |
| Ppp3cb | CGCGTCGTCAAAGCTGTTC | CCTGGGTATCCCATCCATATCAA |
| Ppp3cc | ATGCCACCCCGAAAAGAGG | CATGGTCGGTCCTTCTTGACG |
| Pp4c | CCTTCCCTTTTCCCAGGAGACC | GCCTTGACTTCGCTCTCTTTGA |
| Pp5c | CACTTGTGCGGCAGCGG | CGGCGGTAGTAGCCTTTGAT |
| Pp6c | TGTCACTCCTCCTACCCCAC | GGGCAGCCCTTTAGACCAAT |
| Ppp7ca | TAGAAGTCATGGGCTGTGGC | GCTGGATAGCTGCATTTGGC |
| Ppp7cb | TGTCATCCTGCTCCCTGAGA | CGTATCGAGCATGGAGCTGT |
