# Supplementary material for: PTH stimulation of Rankl transcription is regulated by SIK2 and 3 and mediated by CRTC2 and 3 through action of protein phosphatases 1, 2, 4, and 5
Source: J Biol Chem. 2025 Jul 1;301(8):110434. doi: 10.1016/j.jbc.2025.110434 (PMC12359225; doi:10.1016/j.jbc.2025.110434)
Supplement: Table S3 [file mmc4.docx]

| **Table S3: Antibodies used for Western blotting and immunofluorescence** | | |
| --- | --- | --- |
| **Target** | Antibody | Concentration |
| CRTC 1 | Torc1 Antibody (PA5-17365), Invitrogen | 1:1000 WB  1:250 IF |
| CRTC 2 | CRTC2/TORC2 Antibody (12497-1-AP), Invitrogen | 1:1000 WB  1:200 IF |
| CRTC 3 | Recombinant Anti-CRTC3 antibody [EPR3440] (ab91654) | 1:1000 WB  1:250 IF |
| β-actin | Anti-beta Actin antibody (ab8227), abcam | 1:2000 WB |
| Histone H3 | Histone H3 (D1H2) XP® Rabbit mAb #4499 | 1:3000 WB |
| Lamin B1 | Lamin B1 (D4Q4Z) Rabbit mAb #12586 | 1:3000 WB |
| α-Tubulin | α-Tubulin (Lys40) (D20G3) XP® Rabbit mAb #5335 | 1:3000 WB |
| SIK 1 | Anti-SIK1 antibody (ab217809), abcam | 1:1000 WB  1:200 IF |
| SIK 2 | Recombinant Anti-SIK2 antibody [EPR22722-23] (ab245211), abcam | 1:1000 WB  1:200 IF |
| SIK 3 | Recombinant Anti-SIK3 antibody [EPR23554-71] (ab255701), abcam | 1:1000 WB  1:200 IF |
| PP1 | Polyclonal Rabbit anti‑Human PPP1 [LS‑C406694], LS Bio | 1:1000 WB  1:200 IF |
| PP2 | Recombinant Anti-PP2 [YE351] (ab32065), abcam | 1:1000 WB  1:200 IF |
| PP3 | Recombinant Anti-Calcineurin A antibody [EPR24997-22] (ab282104), abcam | 1:1000 WB  1:200 IF |
| PP4 | PPP4 Rabbit pAb (A13531), ABclonal | 1:1000 WB  1:200 IF |
| PP5 | PPP5 Rabbit pAb (A11712), ABclonal | 1:1000 WB  1:200 IF |
| PP6 | PPP6 Rabbit mAb (A9336), ABclonal | 1:1000 WB  1:200 IF |
| PP7 | Anti-PP7 antibody (ab235601), abcam | 1:1000 WB  1:200 IF |
| Secondary ab (IF) | Goat Anti-Rabbit IgG H&L (Alexa Fluor® 488) (ab150077) | 1:450 IF |
|  |  |  |
